# Identification of Urine Metabolic Biomarkers for Vogt-Koyanagi-Harada Disease

**DOI:** 10.3389/fcell.2021.637489

**Published:** 2021-02-25

**Authors:** Rui Chang, Ying Zhu, Jing Xu, Lin Chen, Guannan Su, Aize Kijlstra, Peizeng Yang

**Affiliations:** ^1^The First Affiliated Hospital of Chongqing Medical University, Chongqing Key Laboratory of Ophthalmology, Chongqing Eye Institute, Chongqing Branch of National Clinical Research Center for Ocular Diseases, Chongqing, China; ^2^University Eye Clinic Maastricht, Maastricht, Netherlands

**Keywords:** Vogt-Koyanagi-Harada disease, metabolomics, urine biomarkers, diagnosis, disease activity

## Abstract

The diagnosis of Vogt-Koyanagi-Harada (VKH) disease is mainly based on a complex clinical manifestation while it lacks objective laboratory biomarkers. To explore the potential molecular biomarkers for diagnosis and disease activity in VKH, we performed an untargeted urine metabolomics analysis by ultra-high-performance liquid chromatography equipped with quadrupole time-of-flight mass spectrometry (UHPLC-Q-TOF/MS). Through univariate and multivariate statistical analysis, we found 9 differential metabolites when comparing VKH patients with healthy controls, and 26 differential metabolites were identified when comparing active VKH patients with inactive VKH patients. Pathway enrichment analysis showed that glycine, serine and threonine metabolism, and arginine and proline metabolism were significantly altered in VKH versus healthy controls. Lysine degradation and biotin metabolism pathways were significantly altered in active VKH versus inactive VKH. Furthermore, the receiver operating characteristic (ROC) curve analysis revealed that the combination of acetylglycine and gamma-glutamylalanine could differentiate VKH from healthy controls with an area under the curve (AUC) of 0.808. A combination of ureidopropionic acid and 5′-phosphoribosyl-5-amino-4-imidazolecarboxamide (AICAR) had an excellent AUC of 0.958 for distinguishing active VKH from inactive VKH. In summary, this study identified abnormal metabolites in urine of patients with VKH disease. Further studies are needed to confirm whether these metabolites are specific for this disease.

## Introduction

Vogt-Koyanagi-Harada (VKH) disease is a multisystemic autoimmune disorder affecting the eye, hair, skin, auditory, and central nervous system (Yang et al., 2007; Sakata et al., 2014). As one of the most common seen uveitis entities in China, it is characterized by a bilateral granulomatous panuveitis (Yang et al., 2005; Du et al., 2016). A combination of autoimmunity, genetic susceptibility, infection, and gut microbiome composition have been suggested as possible etiologic factors of this disease (Weisz et al., 1995; Chi et al., 2007; Greco et al., 2013; Du et al., 2016; Chang et al., 2020; Ye et al., 2020). However, the underlying pathogenesis and exact etiology of VKH disease is still unclear. Diagnosis and monitoring of disease activity of VKH disease is currently based on clinical manifestations while it lacks laboratory biomarkers (Read et al., 2001; Yang P. et al., 2018).

Urinary tests have been applied to diagnose and monitor disease since ancient times (Armstrong, 2007; Dinges et al., 2019). Compared to blood samples, urine offers some specific advantages. First, it is easily accessible and bears no need of invasive sampling. Second, the cost of urinalysis is relatively low. Third, the molecular activity in urine doesn’t change much after sampling which shows a better stability, and it is a relatively clean medium which contains few interfering proteins (Schmidt, 2009; Li et al., 2014; Dinges et al., 2019). Taken together, urine could serve as an ideal and non-invasive specimen to diagnose disease and evaluate disease activity. Little is known about urine metabolite composition in clinical uveitis and this was therefore the purpose of the study presented here.

Metabolomics is a rapidly evolving omics using high-throughput techniques to characterize metabolites in biofluids (Wishart, 2019). The metabolome represents the downstream products of the genome and proteome, finally resulting in a specific omics composition (Yan et al., 2018; Rinschen et al., 2019). It has commonly been used as an approach to identify biomarkers in the diagnosis and prediction of disease (Li et al., 2019; Rinschen et al., 2019; Zhou et al., 2019; Abooshahab et al., 2020; Jiang et al., 2020; Liu Z. et al., 2020; Souto-Carneiro et al., 2020). In view of the unique characteristics of urine, an increasing number of studies have used urinary metabolomics for biomarker discovery in recent years (Kwon et al., 2020; Liu W. et al., 2020; Olesova et al., 2020). Urinary metabolomics has been conducted in several autoimmune diseases, such as rheumatoid arthritis and systemic lupus erythematosus (Alonso et al., 2016). As mentioned above, no urine metabolomic study has been performed in uveitis. In this study we focused on VKH disease, which is a common uveitis entity in China. Untargeted urinary metabolomic analysis was performed to detect putative diagnostic markers. We also compared active with inactive VKH patients to identify potential biomarkers which could distinguish disease activity.

## Materials and Methods

### Participants

The study included 26 VKH patients and 26 healthy controls who were enrolled from the First Affiliated Hospital of Chongqing Medical University (Chongqing, China) between July 2018 and July 2019. Diagnosis of VKH disease was carried out strictly following the diagnostic criteria by the international committee and the modified criteria developed by our team (Read et al., 2001; Yang P. et al., 2018). The exclusion criteria included a history of hypertension, cardiovascular disease, diabetes mellitus, hepatitis, tuberculosis, or other autoimmune diseases such as rheumatoid arthritis, ankylosing spondylitis, systemic lupus erythematosus, and inflammatory bowel disease. None of the patients were treated with topical or systemic corticosteroids, or immunosuppressants for at least 2 weeks prior to urine collection. The 26 enrolled VKH patients included 15 active and 11 inactive VKH patients. Active patients were those with evident inflammatory symptoms in either the anterior or posterior segment, as described elsewhere (Jabs et al., 2005; Chen et al., 2020). Details of the patients are shown in Table 1. The study received the approval of the Ethics Committee of the First Affiliated Hospital of Chongqing Medical University and all participants signed informed consents before collection of urine. All procedures were performed according to the tenets of the Declaration of Helsinki.

**TABLE 1:** Demographic and clinical features of VKH patients and healthy controls.

	VKH (26)		Healthy controls (26)	*p* value
	
	Active VKH (15)	Inactive VKH (11)		
**Demographic**				
Gender (male/female)^a^	10/5	9/2	13/13	0.193
Age (years), median [IQR]^b^	50 [28]	41 [38]	33.5 [19.25]	0.094
BMI (kg/m ^2^), median [IQR]^b^	22.79 [7.99]	21.97 [4.57]	21.18 [6.87]	0.754
**Anterior segment**				
Anterior chamber cells, *n* (%)				
−	0	11 (100)	NA	−
+	8 (53.33)	0	NA	−
++	7 (46.67)	0	NA	−
Keratic precipitates, *n* (%)				
−	1 (6.67)	11 (100)	NA	−
+	2 (13.33)	0	NA	−
++	9 (60)	0	NA	−
+ ++	3 (20)	0	NA	−
Iris nodules (Koeppe/Busacca), *n* (%)	7 (46.67)	2 (18.18)	NA	−
**Posterior segment**				
Choroiditis, *n* (%)	3 (20)	0	NA	−
Retinal detachment, *n* (%)	4 (26.67)	0	NA	−
Optic disk edema, *n* (%)	2 (13.33)	0	NA	−
“Sunset glow” fundus, *n* (%)	10 (66.67)	9 (81.82)	NA	−

*Continuous variables were expressed as median with interquartile range (IQR).*

*^*a*^Analyzed by the Chi-square test.*

*^*b*^Analyzed by the Kruskal-Wallis test.*

*VKH, Vogt-Koyanagi-Harada disease; BMI, body mass index; NA, not applicable.*

### Sample Preparation

Morning urine samples were collected from all participants. The specimens were centrifuged for 30 min (5,000 × *g*, 4°C) to remove debris. The supernatant was stored at −80°C until analyzed. Before analysis, the urine samples were thawed at 4°C, and 100 μL sample was extracted with 400 μL of extraction solvent (methanol: acetonitrile = 1:1). The mixture was vortexed for 30 s, then ultrasound for 5 min, left to stand for 1 h at −20°C, and then centrifuged at 12,000 rpm for 15 min at 4°C. The supernatant was dried in a vacuum concentrator without heating and re-dissolved by adding 100 μL extraction solvent (water: acetonitrile = 1:1) for further ultra-high-performance liquid chromatography equipped with quadrupole time-of flight mass spectrometry (UHPLC-Q-TOF/MS) analysis. We prepared the quality control (QC) samples by mixing 10 μL of each specimen. And then QC samples were applied to analysis together with the tested samples to monitor the stability and repeatability of UHPLC-Q-TOF/MS analysis.

### Metabolomics Analysis

Untargeted urinary metabolomics analysis was performed with an UHPLC system 1,290 (Agilent, United States) with a UHPLC BEH Amide column (2.1 mm × 100 mm, 1.7 μm), coupled with Triple TOF 6600 (AB Sciex, United States) & QTOF 6550 (Agilent, United States). The mobile phase included solvent A (25 mM NH_4_Ac, 25 mM NH_4_OH in water, PH = 9.75) and solvent B (acetonitrile). Elution was as follows: start with 5% solvent A and 95% solvent B for 30 s, decrease to 40% B at 8 min, solvent maintained for 1 min, returned to 95% B for 0.1 min and held for approximately 3 min. The delivery flow rate was 500 μL/min, and 2 μL sample was injected into the column. The Triple TOF mass spectrometer was applied in both positive and negative modes to detect and identify MS/MS spectra on information-dependent acquisition (IDA) during the UHPLC-Q-TOF/MS analysis. The parameters of electrospray ionization (ESI) were set as following: ion source gas 1 = 60 Psi, Ion source gas 2 = 60 Psi, ion spray voltage floating = 5,000 V (+) and −4,000 V (−), curtain gas = 35 Psi and source temperature = 600°C.

The raw data of UHPLC-Q-TOF/MS were processed by R software with XCMS package (version 3.2). The parameters in XCMS were set as minfrac of 0.5 and cutoff of 0.6. After that, we used R package CAMERA for peak annotation. An in-house MS2 commercial database (Biotree Biotech. Co., Ltd., China) was used for metabolite identification. The processed data were applied for further analysis after individual peak filtration, missing value recording, normalization, and integration. Principal component analysis (PCA) and orthogonal projection to latent structures- discriminant analysis (OPLS-DA) was carried out using SIMCA version 16.0.2 (Umetrics AB, Sweden). The variable importance in the projection (VIP) value was calculated to summarize its contribution for each variable in the OPLS-DA model. In addition to multivariate statistical methods, we also performed the Student’ *t*-test to evaluate the significances of metabolites at univariate level. The categories of metabolites were defined by using the Human Metabolome Database (HMDB)^1^.

### Bioinformatics Analysis

Volcano plots were made using GraphPad Prism V.7.0.0 Software, which directly showed the upregulated and downregulated metabolites. MetaboAnalyst^2^ was applied for pathway enrichment analysis and the investigation of altered metabolic pathways.

### Receiver-Operating Characteristic (ROC) Curve Analysis

To identify potential diagnostic biomarkers, ROC curve analysis was used to evaluate the diagnostic potential of differential metabolites and the area under the curve (AUC) was calculated (Zou et al., 2007). To further investigate specific diagnostic biomarkers, a two-step method was performed: (1) differential metabolites were subjected to a stepwise binary logistic regression analysis to establish a combined model; (2) ROC curve analysis was carried out to measure the diagnostic performance of the combined metabolites.

### Statistical Analysis

Statistical analysis was performed using SPSS 22.0 or GraphPad Prism V.7.0.0 Software. The continuous variables of demographic data were assessed by the Shapiro-Wilk test to evaluate whether they were normally distributed. For normally distributed data, we used Student’s t-test to analyze. For analysis of non-normally distributed data, we applied the Kruskal-Wallis test followed by Dunn’s multiple comparison tests. The Chi-square test was used for categorical values. A *p* value less than 0.05 was considered as statistically significant.

## Results

### Urine Metabolism Analysis

**FIGURE 1:**
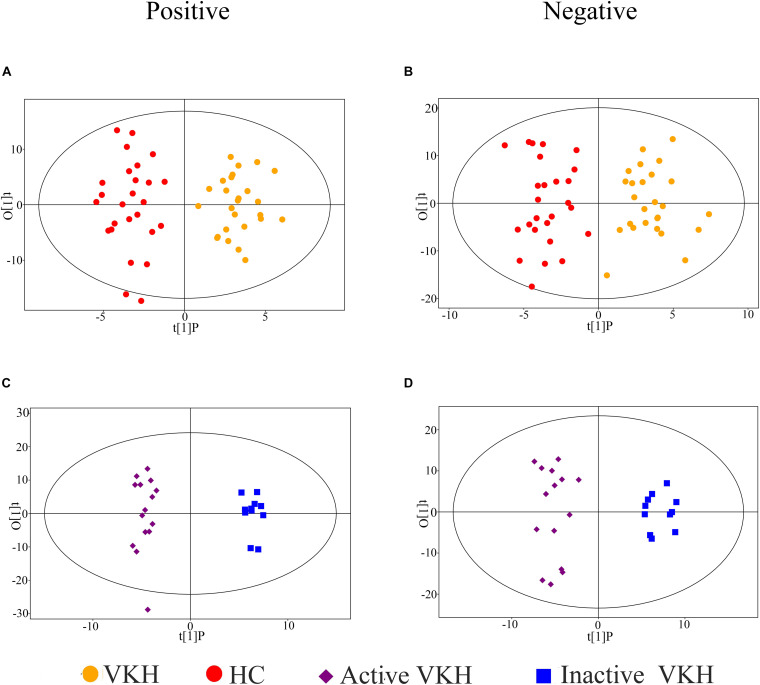
Orthogonal projection to latent structures- discriminant analysis (OPLS-DA) of urinary metabolomics data. OPLS-DA plots for VKH versus healthy controls in positive **(A)** and negative **(B)** ion mode; OPLS-DA plots for active VKH versus inactive VKH in positive **(C)** and negative **(D)** ion mode. VKH, Vogt-Koyanagi-Harada disease; HC, healthy controls.

To investigate the urinary metabolic profiles of VKH disease, we enrolled 26 healthy controls and 26 VKH patients (15 active VKH patients and 11 inactive VKH patients) for untargeted metabolomics analysis. There were no significant differences in gender, age, and body mass index when comparing VKH with healthy controls or active VKH with inactive VKH (Table 1). A total of 2,156 positive-model signals and 2,316 negative-model signals were identified after peak alignment, remodeling of missing values and data normalization (Supplementary Table 1). PCA models were made with the data after log transformation (Supplementary Figure 1). As shown in PCA plots, QC samples were tightly clustered in both positive and negative modes, indicating high reproducibility. OPLS-DA plots were performed to characterize the metabolic profiles of the different groups. As shown in Figure 1, the VKH group was readily distinguished from the healthy controls (Figures 1A,B). Furthermore, a clear separation was seen between active VKH and inactive VKH (Figures 1C,D) in both positive and negative modes.

### Differential Metabolites Analysis of VKH Patients and Healthy Controls

**FIGURE 2:**
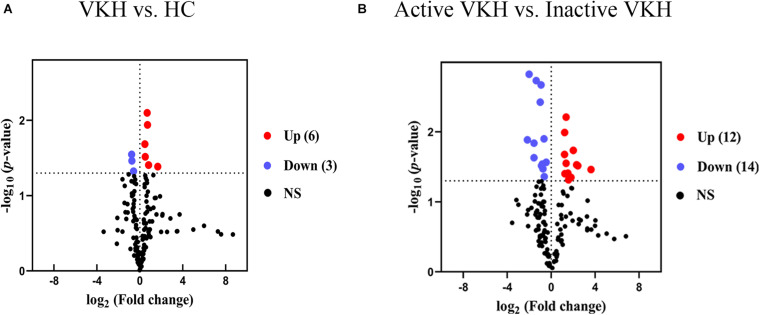
Volcano plots of urinary metabolites. Volcano plots displayed the detected metabolites with log_2_ (fold change) (*x*-axis) and -log_10_ (*p* value) (*y*-axis) when comparing VKH with healthy controls **(A)** and active VKH with inactive VKH **(B)**. The dashed line on *y*-axis indicated *p* value = 0.05. The dashed lines on *x*-axis indicated fold change = 1. The red and blue dots indicate significantly increased and decreased metabolites, respectively. VKH, Vogt-Koyanagi-Harada disease; HC, healthy controls; NS, no significance.

To identify differential metabolite expression between the groups, we applied both multivariate and univariate statistical analyses. In total, 35 metabolites with a VIP > 1 and *p* < 0.05 were considered as differential metabolites (Table 2). Among them, there were 9 differential metabolites for VKH when compared with healthy controls and 26 differential metabolites for active VKH versus inactive VKH. Amino acids were the category with the most abundant differential metabolites. Volcano plots were used to investigate variation tendencies for the differential metabolites (Figure 2). Six metabolites were significantly elevated and 3 metabolites were significantly decreased in VKH as compared to healthy controls (Figure 2A). We also found 12 increased metabolites and 14 decreased metabolites when comparing active VKH with inactive VKH patients (Figure 2B).

**TABLE 2:** Identified differential metabolites.

Metabolites	VKH versus healthy controls			Active versus inactive VKH			ESI ±	Category
		
	VIP	FC	*p* value	VIP	FC	*p* value		
Oxypurinol	3.19	1.65	0.011	/	/	/	+	Purines
gamma-Glutamylalanine	1.15	1.39	0.021	/	/	/	+	Amino acids
Guanidoacetic acid	1.94	0.59	0.028	/	/	/	+	Amino acids
3,4-Dihydroxyhydrocinnamic acid	2.17	0.60	0.034	/	/	/	+	Phenylpropanoids
Sulfapyridine	1.38	3.18	0.041	/	/	/	+	Benzenoids
Tyrosyl-Valine	1.39	0.67	0.047	/	/	/	+	Amino acids
Acetylglycine	2.06	1.60	0.008	/	/	/	−	Amino acids
Phthalic acid	1.03	1.42	0.030	/	/	/	−	Benzenoids
Estrone sulphate	1.12	1.76	0.039	/	/	/	−	Lipids
Ureidopropionic acid	/	/	/	3.29	0.25	0.002	+	Ureas
Biotin	/	/	/	2.98	0.39	0.002	+	Biotin
Phenylalanyl-Aspartate	/	/	/	1.57	0.50	0.004	+	Amino acids
D-Alanyl-D-alanine	/	/	/	1.54	2.56	0.006	+	Amino acids
L-Lysine	/	/	/	1.49	2.34	0.010	+	Amino acids
Histidinyl-Threonine	/	/	/	1.65	0.64	0.013	+	Amino acids
.beta.-Cyano-L-alanine	/	/	/	2.67	0.22	0.013	+	Notmatched^a^
Oxoadipic acid	/	/	/	1.88	0.34	0.015	+	Keto acids
Isovaleric acid	/	/	/	1.42	2.32	0.021	+	Lipids
Androstenedione	/	/	/	3.49	0.34	0.023	+	Lipids
Acetylcysteine	/	/	/	1.75	0.73	0.027	+	Amino acids
L-Pipecolic acid	/	/	/	1.27	2.57	0.028	+	Amino acids
Threoninyl-Threonine	/	/	/	2.24	4.97	0.030	+	Amino acids
Serylaspartic acid	/	/	/	1.00	0.59	0.034	+	Amino acids
L-Leucine	/	/	/	1.57	0.60	0.034	+	Amino acids
1-Myristoyl-sn-glycero-3-phosphocholine	/	/	/	2.59	12.39	0.035	+	Notmatched^a^
2-Hydroxybutyric acid	/	/	/	1.36	2.89	0.039	+	Hydroxy acids
D-Glucuronic acid	/	/	/	1.79	2.39	0.040	+	Carbohydrates
Alanyl-Threonine	/	/	/	1.25	0.65	0.044	+	Amino acids
Phenyllactic acid	/	/	/	1.24	3.51	0.045	+	Phenylpropanoids
Indolelactic acid	/	/	/	1.38	3.00	0.049	+	Indoles
5′-phosphoribosyl-5-amino-4-imidazolecarboxamide	/	/	/	1.15	0.53	0.002	−	Nucleosides
2′-*O*-Methyluridine	/	/	/	1.81	4.05	0.018	−	Not matched^a^
2-Ketohexanoic acid	/	/	/	1.16	0.57	0.029	−	Keto acids
N1-Methyl-2-pyridone-5-carboxamide	/	/	/	1.08	0.54	0.030	−	Pyridines
Maleic acid	/	/	/	1.47	5.38	0.031	−	Dicarboxylic acids

*VKH, Vogt-Koyanagi-Harada disease; VIP, variable importance in the projection; FC, fold change; ESI, electrospray ionization.*

*^*a*^The detected metabolite could not be matched to a known metabolite in Human Metabolome Database. *P* value was calculated by Student’s *t*-test.*

### Pathway Analysis of Differential Metabolites

**FIGURE 3:**
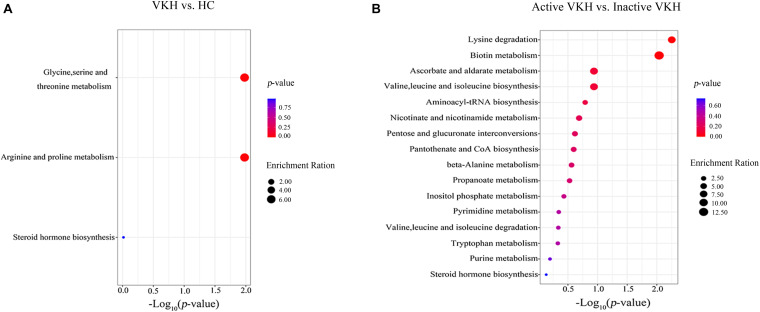
Pathway enrichment analysis of differential metabolites. Pathway enrichment analysis of differential metabolites identified in VKH versus healthy controls **(A)** and in active VKH with inactive VKH **(B)**. The *x*-axis represented the *p* value and the y-axis represented the pathways. The color of dots indicates pathway enrichment significance. The size of dots indicates pathway impact. VKH, Vogt-Koyanagi-Harada disease; HC, healthy controls.

MetaboAnalyst was used to investigate urine metabolite changes in VKH versus healthy controls and active VKH versus inactive VKH (Figure 3). The significantly altered metabolic pathways are summarized in Supplementary Table 2. When comparing VKH with healthy controls, glycine, serine and threonine metabolism, and arginine and proline metabolism were significantly altered metabolic pathways. Both these two pathways were enriched by decreased guanidoacetic acid in VKH disease (Figure 3A). Lysine degradation and biotin metabolism pathways were significantly altered in active VKH as compared to inactive VKH (Figure 3B).

### Diagnostic Biomarkers From Urine for VKH Disease

**FIGURE 4:**
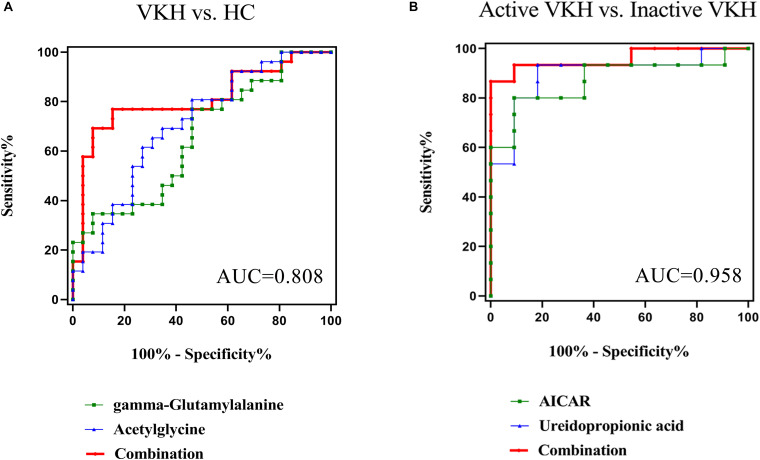
Receiver-operating characteristic (ROC) curve analysis. The ROC curves based on the combination of differential metabolites for VKH versus healthy controls generated an AUC of 0.808 **(A)**, and active VKH versus inactive VKH generated an AUC of 0.958 **(B)**. VKH, Vogt-Koyanagi-Harada disease; HC, healthy controls; AUC, area under the curve.

To investigate possible diagnostic biomarkers for VKH disease, ROC analyses were performed to evaluate the diagnostic power of each of the identified 9 differential metabolites (Supplementary Table 3). All the 9 differential metabolites yielded a diagnostic ability with an AUC > 0.5 and only acetylglycine had an AUC > 0.7 for distinguishing VKH disease from healthy controls. To explore more effective diagnostic biomarkers, we used the stepwise binary logistic regression analysis to establish a combined model. The model revealed a combination of an increased acetylglycine and gamma-glutamylalanine to be specific for VKH disease, equated as Logit (P) = −3.244 + 0.064^∗^acetylglycine + 0.023^∗^gamma-glutamylalanine. The combination of acetylglycine and gamma-glutamylalanine generated an AUC at 0.808 (Figure 4A).

### Disease Activity Urine Biomarkers for VKH Disease

When comparing active with inactive VKH we found 26 differential urine metabolites. ROC analyses showed that all 26 metabolites had an AUC > 0.7 (Supplementary Table 3). Through the stepwise binary logistic regression analysis, we found that a predictive model consisting of ureidopropionic acid and 5′-phosphoribosyl-5-amino-4-imidazolecarboxamide (AICAR) could differentiate active VKH from inactive VKH, equated as Logit (P) = 8.106-0.012^∗^ureidopropionic acid-0.538^∗^AICAR. The ROC curve analysis showed that the combination of a decreased ureidopropionic acid and AICAR yielded an excellent AUC of 0.958 (Figure 4B).

## Discussion

In the present study, we found a urine metabolite signature in patients with VKH disease. A total of 35 differential metabolites including nine metabolites for VKH were observed when compared with healthy controls and 26 metabolites for active versus inactive VKH. Among them, amino acids were the most abundant differential metabolite category. We further identified 2 altered pathways including glycine, serine and threonine metabolism, and arginine and proline metabolism when comparing VKH patients with healthy controls. Lysine degradation and biotin metabolism pathways were found to be significantly altered in active VKH when compared with inactive VKH. Furthermore, a diagnostic biomarker panel consisting of acetylglycine and gamma-glutamylalanine could distinguish VKH from healthy controls with an AUC of 0.808. The combination of ureidopropionic acid and AICAR was identified as a predictive biomarker with an excellent AUC of 0.958, which could discriminate active VKH from inactive VKH.

To the best of our knowledge this is the first study reporting urinary metabolomics in uveitis. Previous studies addressed aqueous humor or plasma as a source for metabolomics analysis Possner Schlossman syndrome and HLA-B27 associated acute anterior uveitis (Verhagen et al., 2019; Wang et al., 2019). Although aqueous humor is a biological fluid in direct contact with the site of inflammation it is an invasive procedure and ethical considerations may limit its use. Several uveitis entities, including VKH, also have a systemic involvement and disease activity might be reflected by urine metabolite composition. The current study on urine is an extension of our earlier metabolomics studies in VKH where we used sweat and plasma as a biological source (Chen et al., 2020; Cui et al., 2020). In this study we revealed significant differences in urine metabolite composition between VKH patients and healthy controls or between active VKH and inactive VKH patients. Urine offers many advantages over other body fluids. For example, it can be longitudinally collected and is non-invasive. Urine contains a large variety of metabolites, mostly following filtration of blood in the kidneys and many routine clinical assays are performed on this fluid. In the field of current uveitis management, urine analysis does not have a prominent place. We believe that modern technology using metabolomics will change this attitude in the near future.

The diagnosis of VKH disease is challenging because of the complicated clinical manifestations and unclear etiology. The investigation of diagnostic biomarkers and disease activity biomarkers could contribute to the diagnosis and might be useful to predict the clinical stage of the disease. Acetylglycine and gamma-glutamylalanine both belong to the amino acid pathways and may be involved in immunoregulation (Bonvini et al., 2018; Yang X. et al., 2018; Kelly and Pearce, 2020). Acetylglycine can be generated by acetylation of glycine (Polevoda and Sherman, 2002). Glycine was reported to exert immunomodulatory effects in many immune cells including macrophages and T lymphocytes (Wheeler et al., 1999; Xu et al., 2008; Van den Eynden et al., 2009). Gamma-glutamylalanine is a dipeptide composed of gamma-glutamate and alanine. Gamma-glutamate has been shown to prevent the development of atopic dermatitis through the modulation of the Th2 immune response (Lee et al., 2014). Taken together, these results suggest a possible role of amino acid metabolism in the pathogenesis of VKH disease.

Previous metabolomic studies have shown an association between a disturbance of amino acid metabolism and autoimmune disease and a certain amino acid profile might have a diagnostic potential (Fernández-Ochoa et al., 2019; Thoman and McKarns, 2020; Yan et al., 2020). Similarly, we found that amino acids were differentially expressed in urine from VKH patients and they were the most abundant differential metabolites in the present study. Our study is in agreement with a recent study in rheumatoid arthritis showing that serum guanidoacetic acid could serve as a prognostic biomarker for disease activity (Sasaki et al., 2019). Guanidoacetic acid is biosynthesized from glycine by glycine amidinotransferase (Sasaki et al., 2019). Pathway enrichment analysis showed that the altered glycine, serine and threonine metabolism and arginine and proline metabolism were evidenced by decreased guanidoacetic acid in VKH disease. Our previous plasma metabolomics study also revealed a dysregulated glycine, serine and threonine metabolism in VKH disease (Chen et al., 2020). These findings suggest that glycine, serine, and threonine metabolism may play a role in the immunopathogenesis of VKH disease. Lysine as an essential amino acid was shown to be significantly elevated in feces obtained from patients with inflammatory bowel diseases (Marchesi et al., 2007). In this study, we found that both altered lysine degradation and biotin metabolism were involved as shown by the increased urinary L-lysine level in active VKH as compared with inactive VKH. This suggest that an increased L-lysine might contribute to immune activation in VKH disease. Future study is needed to validate the exact role of the concerned differential metabolites and related pathways identified in this study in the pathogenesis of VKH disease.

Accurate assessment of disease activity may optimize the therapeutic regimen in our patients with intraocular inflammation. However, assessment of disease activity of VKH disease is currently based on clinical manifestations which lack of objective laboratory biomarkers. In the present study, we showed that the combination of ureidopropionic acid and AICAR in urine could serve as a predictive biomarker to differentiate active from inactive VKH. In a previous study we failed to find any metabolic difference in the plasma between active and inactive VKH (Chen et al., 2020). The reason as to why there is a discrepancy between urine and plasma is not clear. A longitudinal study on metabolites using a large patient sample size is needed to address this issue.

Our study has a number of limitations. As we only enrolled VKH patients and healthy controls, it cannot be concluded that the abnormal metabolites identified in this study are specific for VKH disease. More studies are needed to clarify this issue in the future, including other uveitis entities. The sample size used in this study is relatively small, mostly due to the difficulty in collecting samples from active VKH patients not yet receiving treatment. As mentioned above, further longitudinal studies are needed with larger numbers of patients to study differences concerning urine and blood metabolites in VKH patients at different stages of this disease.

In conclusion, the study showed that the combination of urinary acetylglycine and gamma-glutamylalanine was able to discriminate VKH disease from healthy controls. A biomarker panel consisting of ureidopropionic acid and AICAR had a satisfactory performance for the discrimination of VKH disease activity. The current study shows that the application of urine metabolite analysis may serve as an auxiliary diagnostic tool and be helpful for the study of the pathophysiological mechanisms involved in the development of VKH disease.

## Data Availability Statement

The original contributions presented in the study are included in the article/Supplementary Material, further inquiries can be directed to the corresponding author/s.

## Ethics Statement

The studies involving human participants were reviewed and approved by The Ethics Committee of the First Affiliated Hospital of Chongqing Medical University. The patients/participants provided their written informed consent to participate in this study.

## Author Contributions

RC and PY conceived the idea and designed the experiments. RC, YZ, and LC collected the sample. RC and LC performed all the experiments. RC, YZ, and JX analyzed the data, wrote the manuscript. PY, GS, and AK interpreted data and revised the manuscript. All authors contributed to the article and approved the submitted version.

## Conflict of Interest

The authors declare that the research was conducted in the absence of any commercial or financial relationships that could be construed as a potential conflict of interest.

## Footnotes

1 https://hmdb.ca/

2 https://www.metaboanalyst.ca/
