# Supplementary material for: Identification of Urine Metabolic Biomarkers for Vogt-Koyanagi-Harada Disease
Source: Front Cell Dev Biol. 2021 Feb 25;9:637489. doi: 10.3389/fcell.2021.637489 (PMC7947328; doi:10.3389/fcell.2021.637489)
Supplement: Supplementary file 1 [file Table_1.DOCX]

**Figure S1.** Principal component analysis (PCA) of urinary metabolomics


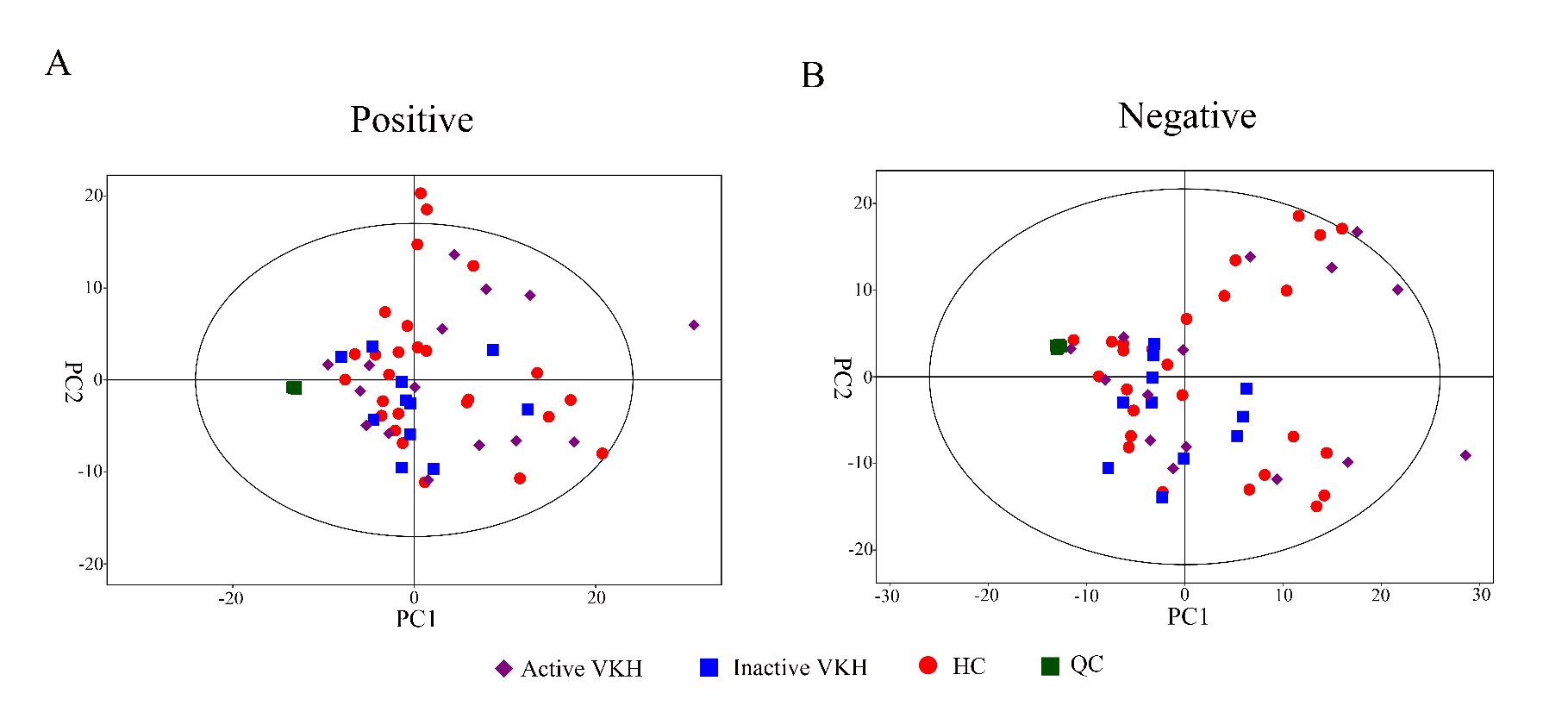


PCA of urinary metabolomics data in positive model (A) and negative model (B).

VKH: Vogt-Koyanagi-Harada disease, HC: healthy controls, QC: quality control.

**Table S1** (please see attached excel file)

**Table S2.** The significantly altered metabolic pathways.

|  | **Pathways** | ***p* value** | **Hit** | **Metabolites** |
| --- | --- | --- | --- | --- |
| VKH versus healthy control | Glycine, serine and threonine metabolism | 0.01 | 1 | Guanidoacetic acid |
|  | Arginine and proline metabolism | 0.01 | 1 | Guanidoacetic acid |
| Active VKH versus Inactive VKH | Lysine degradation | 0.006 | 3 | L-Lysine, Oxoadipic acid, L-Pipecolic acid |
|  | Biotin metabolism | 0.009 | 2 | Biotin, L-Lysine |

VKH: Vogt-Koyanagi-Harada disease.

**Table S3.** Area under the curve (AUC) values obtained through receiver-operating characteristic (ROC) analyses of 35 differential metabolites.

| **Metabolites** | **VKH versus**  **healthy control** | **Active VKH versus**  **Inactive VKH** |
| --- | --- | --- |
| Acetylglycine | 0.709 | / |
| 3,4-Dihydroxyhydrocinnamic acid | 0.689 | / |
| Oxypurinol | 0.685 | / |
| Guanidoacetic acid | 0.682 | / |
| gamma-Glutamylalanine | 0.655 | / |
| Phthalic acid | 0.642 | / |
| Tyrosyl-Valine | 0.638 | / |
| Estrone sulfate | 0.601 | / |
| Sulfapyridine | 0.55 | / |
| Ureidopropionic acid | / | 0.897 |
| 5'-phosphoribosyl-5-amino-4-imidazolecarboxamide | / | 0.873 |
| Biotin | / | 0.867 |
| D-Alanyl-D-alanine | / | 0.848 |
| .beta.-Cyano-L-alanine | / | 0.824 |
| Phenylalanyl-Aspartate | / | 0.818 |
| 2-Hydroxybutyric acid | / | 0.782 |
| 2'-O-Methyluridine | / | 0.782 |
| Histidinyl-Threonine | / | 0.77 |
| Androstenedione | / | 0.764 |
| Threoninyl-Threonine | / | 0.764 |
| Oxoadipic acid | / | 0.758 |
| D-Glucuronic acid | / | 0.758 |
| L-Pipecolic acid | / | 0.739 |
| Phenyllactic acid | / | 0.739 |
| N1-Methyl-2-pyridone-5-carboxamide | / | 0.739 |
| L-Lysine | / | 0.733 |
| Indolelactic acid | / | 0.733 |
| Isovaleric acid | / | 0.727 |
| L-Leucine | / | 0.727 |
| 2-Ketohexanoic acid | / | 0.727 |
| Alanyl-Threonine | / | 0.723 |
| Acetylcysteine | / | 0.721 |
| Serylaspartic acid | / | 0.715 |
| Maleic acid | / | 0.636 |
| 1-Myristoyl-sn-glycero-3-phosphocholine | / | 0.608 |

VKH: Vogt-Koyanagi-Harada disease.
